# Effects of a Walking-Based Physical Activity Intervention on Health Indicators in University Students: Protocol for a Randomized Controlled Trial

**DOI:** 10.2196/83983

**Published:** 2025-12-10

**Authors:** Andrés Godoy-Cumillaf, Paola Fuentes-Merino, Frano Giakoni-Ramírez, Catalina Muñoz-Strale, Josivaldo de Souza-Lima, Daniel Duclos-Bastías, Maribel Parra-Saldías, José Bruneau-Chávez, Eugenio Merellano-Navarro

**Affiliations:** 1 Grupo de Investigación en Educación Física, Salud y Calidad de Vida (EFISAL), Facultad de Educación Universidad Autónoma de Chile Temuco, Araucanía Chile; 2 Facultad de Educación y Ciencias Sociales, Instituto del Deporte y Bienestar Universidad Andres Bello Santiago de Chile Chile; 3 iGEO Group, School of Physical Education, Faculty of Philosophy and Education Pontificia Universidad Católica de Valparaíso Valparaíso Chile; 4 METIS Research Group, Facultad de Negocios y Tecnología Universidad Alfonso X el Sabio (UAX) Madrid Spain; 5 Departamento de Educación Física, Deporte y Recreación Universidad de Atacama Copiapó, Atacama Chile; 6 Departamento de Educación Física, Deportes y Recreación Universidad de la Frontera Temuco, Araucania Chile; 7 Department of Physical Activity Sciences, Faculty of Education Sciences Universidad Católica del Maule Talca Chile

**Keywords:** body composition, cardiorespiratory fitness, fat, muscle strength, pedometer

## Abstract

**Background:**

Regular participation in some type of physical activity brings improvements in health indicators such as cardiorespiratory fitness, muscle strength, and body composition. However, despite evidence indicating health benefits, 1 in 4 adults is physically inactive, a situation that also occurs in the university population. Walking is a physical activity modality that can be easily incorporated into daily activities; therefore, using a walking-based physical activity intervention could improve some health indicators.

**Objective:**

This protocol aims to analyze the impact of a walking-based physical activity intervention on health indicators in university students.

**Methods:**

An intervention group (n=99) and a control group (n=99) will be randomly selected. All participants will be assessed at the beginning and end of the intervention for indicators of health, cardiorespiratory fitness, muscle strength, and body composition. The intervention group will participate in a 14-week walking program with individualized daily goals, self-monitoring, personalized feedback, and weekly educational material, while the control group will only record their steps without receiving personalized goals or feedback.

**Results:**

The recruitment process will begin in March 2026. Initial assessments are scheduled to take place from March 2, 2026, to March 13, 2026. The intervention will be performed from March 16, 2026, to June 19, 2026 (14 weeks). From June 22, 2026, to July 6, 2026, the final evaluations will be performed. The final results of this study are expected to be published by October 2026.

**Conclusions:**

This protocol proposes a novel and feasible approach to overcome common barriers to physical activity in university students, with the potential for large-scale application in similar contexts.

**Trial Registration:**

ClinicalTrials.gov NCT06580769; https://clinicaltrials.gov/study/NCT06580769

**International Registered Report Identifier (IRRID):**

PRR1-10.2196/83983

## Introduction

### Background

Regular participation in some form of physical activity brings a variety of benefits to those who engage in it [1], including improvements in health indicators, such as cardiorespiratory fitness [2,3], muscle strength [4,5], and body composition [6]. However, despite evidence demonstrating its benefits, 1 in 4 adults is physically inactive [7], as they do not meet the recommended duration of 150 to 300 minutes of moderate physical activity per week or 75 to 150 minutes of vigorous physical activity [8]. Among the elements that explain this situation are motivation, access to sports centers, or the support of family or society, which ultimately lead to adults avoiding participation in physical activity [9,10]. In the university population, the situation is similar, as academic pressure is added to other obligations inherent in their lives, resulting in the adoption of unhealthy habits [11,12]. Thus, evidence shows that university students spend 9.82 hours a day in a sedentary state (a value that has been increasing over the last 10 years), which is considerably higher compared to the general population of young adults, a situation that is associated with a higher risk of harmful health outcomes [13].

One means that has been shown to be useful in increasing participation physical activity interventions [14] have been shown to be useful in increasing participation in physical activity and thus reducing health risks, with the most promising being personalized interventions [15]. One option is the measurement of the number of daily steps—a method that has been shown to have the potential to cause increases in physical activity because it is a modality that can be easily incorporated into daily activities [16]—being a valid alternative to meet physical activity recommendations because evidence suggests that moderate to vigorous intensity physical activity recommendations can be achieved, on average, with a total volume of 10,000 steps/d for adults [17]. Recent research implementing physical activity interventions based on daily step measurement has used wearable daily step trackers as a means of monitoring, as they have the facility to allow users to use them in conjunction with mobile phones, allowing them to immediately track their activity and thus manage their time to improve their health [18]. Thus, physical activity interventions based on daily step measurement have been implemented in university students using step trackers, which have demonstrated increases in minutes per day of moderate to vigorous intensity physical activity [19,20]. However, despite having been shown to be efficient in increasing participation, most studies are short term, usually lasting 7 days [21], and studies using longer periods are still limited in terms of evidence on their efficacy [22,23].

As can be seen and as explained by Preusse et al [24], measuring daily steps can provide support to those looking to increase their levels of physical activity. Step trackers are a recommended choice because they are inexpensive and easy to use and provide personalized, ongoing support [16].

Given that adults exhibit low compliance with physical activity recommendations, in particular the university population, which is in a period where daily time is prioritized in their academic activities, the performance of a physical activity intervention based on increasing the number of daily steps and monitored through step trackers represents a potential way to overcome the barriers that hinder the practice of physical activity. In addition, given that the use of this technology provides personalized support, it is reasonable to hypothesize that performing a physical activity intervention that increases the number of daily steps could improve some health indicators.

### Objective

The objective of this study is to provide a working methodology that allows for the analysis of the impact of a walking-based physical activity intervention on health indicators in university students.

## Methods

### Design

It will be a 2-arm randomized controlled trial (RCT) to analyze a physical activity intervention in college students for 14 weeks. The RCT will be reported according to the CONSORT (Consolidated Standards of Reporting Trials) statement [25] and the SPIRIT (Standard Protocol Items: Recommendations for Interventional Trials) guidelines [26] (Multimedia Appendix 1).

### Participants

Participants will be gathered from the Universidad Autónoma de Chile across the campuses located in the cities of Santiago, Talca, and Temuco. Information about the intervention will be disseminated through institutional email, social networks, and informative posters. The following inclusion criteria will be considered: (1) be a university student with active enrollment at the Universidad Autónoma de Chile, (2) aged between 18 and 25 years, (3) have medical consent that authorizes the regular practice of physical activity, and (4) sign an informed consent form to participate in the study. The exclusion criteria will be as follows: (1) having a physical or mental impediment that prevents or limits the performance of physical activity and (2) belonging to special groups, such as professional athletes or pregnant women. The evaluations at the beginning and end of the study will be performed at the university’s facilities using the equipment available at each of the respective campuses (Santiago, Talca, and Temuco).

### Randomization

This study will adopt a single-blind RCT design. Using EPIDAT software (version 4.2; Pan American Health Organization), participants will be divided into 2 groups (ie, group 1 [intervention] and group 2 [control]).

### Sample Size Calculation

On the basis of what was proposed for the design of the RCTs [27], the number of participants will be calculated. Taking 2 RCTs that aimed to increase physical activity levels through walking [18,19] as reference, the sample will be calculated considering an absolute difference of 3% (α error of .05 and statistical power of .80) between the intervention group and the control group in the mean percentage of cardiorespiratory endurance (SD 5), giving a required sample of 99 participants for each group (intervention and control).

### Intervention

#### Training Intervention

For step monitoring, a wearable Xiaomi smart bracelet step tracker (*Smart Band 9 Active* model), validated for step tracking, will be used on the nondominant wrist for at least 12 hours a day [28]. Participants will receive physical activity goals based on adherence to international recommendations for steps per day (≥10,000 steps/d) [17,29,30].

**Figure 1:**
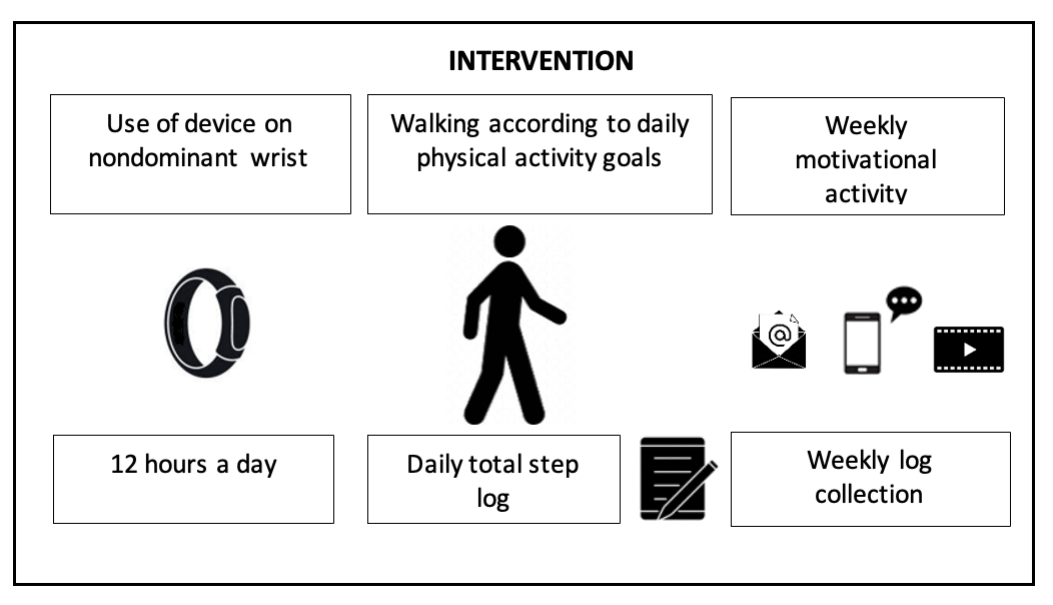
Group intervention activities.

A member of the research team will provide personalized feedback via email or WhatsApp (Meta Platform, Inc) daily, reinforcing achievements and adjusting goals when necessary. In addition, educational infographics designed to promote physical activity will be sent weekly, detailing the benefits of walking, strategies to increase the step counts, and practical recommendations for maintaining adherence (Figure 1). The weekly and total step records will be obtained through a downloadable CSV file from the Mi Fitness app (Xiaomi Mobile Software Co, Ltd), which will be downloaded by the user and sent to the research team via email.

#### Duration of the Intervention

**Figure 2:**
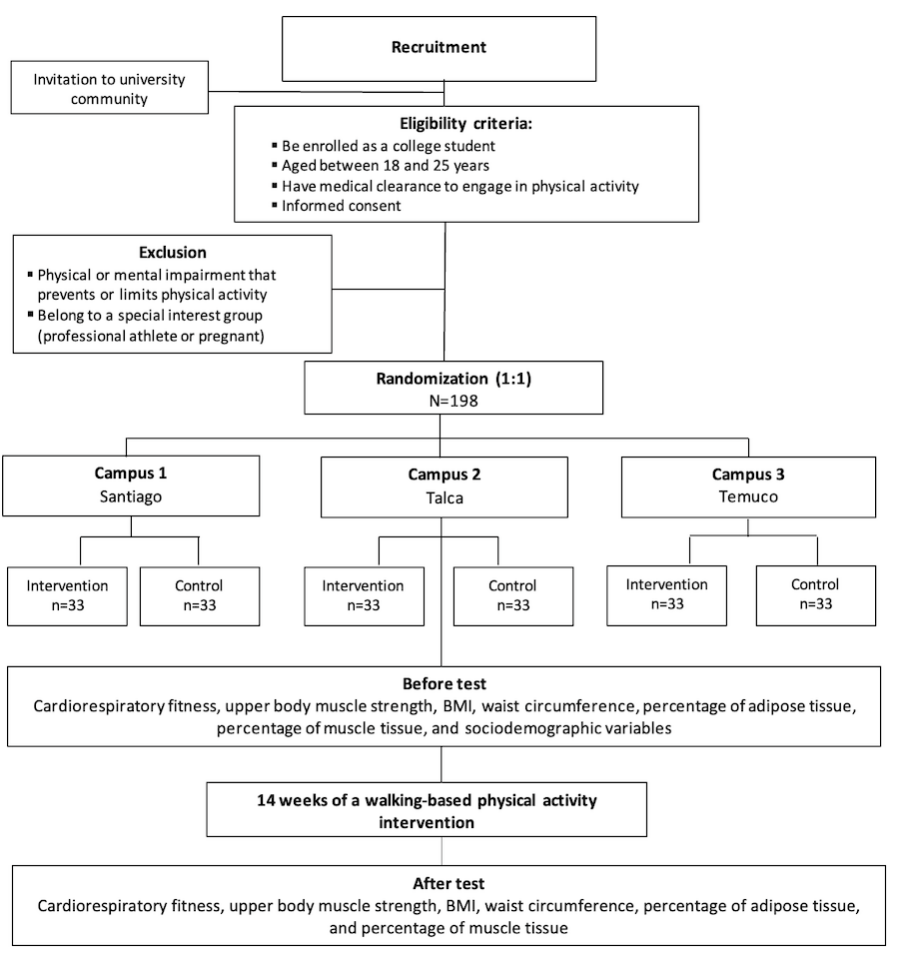
Recruitment, randomization, and evaluation flowchart.

The intervention will last 14 weeks. Initial assessments are scheduled to take place from March 2, 2026, to March 13, 2026. The intervention itself will be performed from March 16, 2026, to May 22, 2026. Final evaluations will be performed from May 25, 2026, to June 5, 2026. Inclusion and exclusion criteria, participant selection and randomization procedures, as well as baseline and follow-up assessments are summarized in the flowchart presented in Figure 2.

#### Adherence to the Intervention Criteria

Those who make up the control group will also have their health indicators evaluated at the beginning and end of the intervention. Participants will also use the same *Xiaomi Smart Band 9* Active for at least 12 hours a day and will submit the weekly and total step log report, downloaded from the Mi Fitness app, to the research team. However, they will not receive specific step goals, individualized feedback, or behavior change strategies. They will maintain their usual physical activity habits without setting quantitative targets or providing personalized motivational messages.

### Procedures

Signed informed consent will be obtained once the sample has been recruited. Then, the evaluations of cardiorespiratory endurance, muscle strength, body composition, and the sociodemographic questionnaire will be scheduled. Subsequently, each participant will be given a step tracking device as well as training on their operation, maintenance, and how to download the step report from the Mi Fitness app.

### Measuring Health Indicators

#### Cardiorespiratory Fitness

This indicator will be measured through the 20-m shuttle run test [31]. Participants must run between 2 lines separated by 20 m. The speed at which they must move is indicated by a sound emitted by a recorded tape. The participant begins at a speed of 8.5 km/h, which increases by 0.5 km/h every minute. When a participant being evaluated fails to reach the lines twice in a row, along with the sound, or when they are unable to continue running the test, the evaluation is completed, and the minute reached is recorded. According to the proposal, the maximum oxygen consumption is estimated [31].

#### Upper Body Muscle Strength

Using a digital dynamometer (Takei T.KK.5401; Takei Scientific Instruments Co, Ltd), grip strength will be evaluated. For each hand, the average of 2 measurements will be calculated.

#### Body Composition

Using a digital scale and a stadiometer, weight and height will be measured (Tanita MC-780U [Tanita Corporation] and Seca 222 [Seca, GmbH & Co], respectively). For weight, the participant must be without shoes and with as little clothing as possible, while for height, they must be in a standing position, obtaining the value at the end of a normal inspiration. Both measurements will be taken in duplicate using the average to calculate the BMI.

Waist circumference will be determined as mentioned subsequently. On the right side of the body, a mark will be made on the skin at the height of the last rib, then a mark will be made on the skin at the top of the iliac crest. The distance between both marks will be calculated, and, at the midpoint, a final mark will be made. The same procedure will be performed on the left side. Using a tape measure, which must be at the height of both end marks on each side, the value will be determined at the end of a normal expiration. For the analyses, the average of 2 measurements will be used.

The percentage of adipose and muscle tissue will be calculated from the average of 2 evaluations performed in a state of rest and fasting, with the minimum amount of clothing and after urinating and resting for 15 minutes, using tetrapolar electrical bioimpedance (Tanita MC-780U). It will be complemented by the measurement of skinfolds, body perimeters, and bone diameters [32].

### Covariables

Before the start of the intervention, sociodemographic variables will be collected through a questionnaire, including questions on age, sex, career, means of transport, geographic area of residence, screen time, and socioeconomic level of the participants.

### Ethical Considerations

This protocol was approved by the scientific ethics committee of the Universidad Autónoma de Chile as well as the informed consent document (Scientific Ethics Committee CECP 28-25), which participants must sign at the beginning of the study. They will receive a copy of the signed consent form and will be given the opportunity to ask questions related to the study. The procedures will be executed in accordance with the Declaration of Helsinki. In addition, this study has been registered on ClinicalTrials.gov (NCT06580769). Participants may withdraw from the study at any time without having to justify their decision. To safeguard participant privacy and the integrity of the collected data, the following measures will be implemented: the collected data are confidential and will be stored in locked files accessible only to the principal investigator. The data will be anonymized. Only anonymized data will be used for publications or presentations.

### Statistical Analysis

The normality of the data will be checked through the Kolmogorov-Smirnov test, which will indicate the steps to be taken in relation to the comparison tests. Once the pretest tests have been performed, comparison analyses will be performed between groups (2-tailed Student *t* test or Mann-Whitney *U* test) to check the homogeneity of the groups. At the conclusion of the final evaluations of the study, intragroup differences will be analyzed using a statistical test of repeated measures (Student *t* test for related samples or Wilcoxon signed-rank test), along with the intergroup differences (Student *t* test or Mann-Whitney *U* test).

Cohen *d* will be used to determine the effect size on variables in which significant differences are found, using the following scale: 0.2 or greater=small, 0.5 or greater=moderate, and 0.8 or greater=large [33]. In addition, regression analyses will be performed incorporating the covariates. The simple imputation method will be used for all analyses [34], keeping the participants in the group to which they were assigned (intervention or control) regardless of the sessions they attended [35]. In valid cases, analyses will be performed to determine sensitivity [36]. For all tests, statistical significance will be set to *P*<.05. The data will be analyzed using SPSS statistics (version 29; IBM Corp).

## Results

The project was awarded in April 2025. Subsequently, the project was reviewed for possible updates. In August 2025, funds were received, and the necessary materials for the execution of the project were purchased. Between September 2025 and December 2025, the staff in charge of the evaluations will be trained. The recruitment process will begin in March 2026. Initial assessments are scheduled to take place from March 2, 2026, to March 13, 2026. The intervention will be conducted from March 16, 2026, to June 19, 2026 (14 weeks). From June 22, 2026, to July 6, 2026, final evaluations will be conducted. The final results of the study are expected to be published by October 2026. The main results will be provided to the participants in a summarized way and will also be disseminated to the scientific community through participation in scientific events.

## Discussion

### Anticipated Findings

Considering that there is currently a high number of inactive adults [7] and that sedentary behaviors predominate in university students’ daily lives due to academic demands—which is associated with damage to their health [13]—it is necessary to implement strategies to help address this problem. Interventions based on wearable technologies have shown increases in daily step counts and, to a lesser extent, activity intensity, with small to moderate effects and heterogeneity between populations [16,18,26]. These findings support the use of goals and objective monitoring as key components.

One option that has proven to be effective, low cost, and easy to implement is physical activity through walking, which is monitored by daily step trackers. However, most studies implementing this strategy have generated evidence by monitoring physical activity for 7 days on average [37], which could cause the reported results to be biased because participants could change their physical activity behaviors because they are aware that they are being evaluated [21]. This reinforces the need for research with longer follow-up periods that allow for a more detailed analysis of the relationship between daily steps and health indicators. In the university population, recent studies have shown that programs of 8 to 12 weeks supported by mobile apps and messaging can lead to significant increases in daily steps [21,22,25,27]. On the basis of this evidence, this protocol proposes a duration of 14 weeks, with individualized goals and weekly feedback, with the aim of achieving more sustained effects than those observed in interventions of shorter duration.

The choice of a long period is based on the need to assess the sustainability of change, given that recent reviews suggest that initial increases may decrease over time [5,20]. Likewise, setting explicit daily goals has been associated with higher levels of compliance, and more recent proposals even propose hourly objectives as a tactic to achieve the daily goal [17,29].

Regarding the results, cardiorespiratory fitness, muscle strength, and body composition were included because of their value as relevant clinical markers. Current evidence shows that higher fitness levels in youth are associated with better indicators of cardiometabolic health and overall well-being [13,18]. In addition, studies have linked muscle condition with a better perception of health in adolescents [5], which reinforces the relevance of these indicators as outcomes of this study.

Methodologically, the use of a validated wrist device, combined with daily manual logging, addresses both ethical considerations of data privacy and the intention of encouraging self-regulation and awareness of participants’ own physical activity [19]. This component has been described as an effective behavior change strategy in digital interventions [16,18]. Likewise, the risk of reactivity to initial monitoring is recognized [21,38]; therefore, extended duration seeks to minimize this effect and allow a more stable analysis of behavior over time.

The implementation of this study and the subsequent results could be useful for the organizations in charge of providing comprehensive well-being to university students, offering programs that are not only dedicated to their professional training but also contribute to improving their health. This research also aligns with Sustainable Development Goal 3, target 3.4 [39], which aims to promote more active lives and contributes to reducing premature mortality from non-communicable diseases, along with promoting mental health and well-being [37].

The strength of this research is that the intervention will be based on walking, which is an easy and low-cost activity to perform because it does not need additional implements to those used daily by a person nor does it require sports facilities. The device used to track the steps is low cost, so it is accessible to anyone; its use can be constant throughout the day because it does not cause major discomfort when used as a watch that adapts to the characteristics of the wrist, in addition to providing information on physical activity of better quality than that collected through self-reports.

### Conclusions

In conclusion, this protocol describes a feasible, low-cost approach to evaluating the impact of a walking intervention on the health of college students. The 14-week design, supported by wearable technologies, individualized goals, and personalized feedback, seeks to overcome the limitations of short-term studies and provide useful evidence for health promotion in the university setting.

## Appendix

Multimedia Appendix 1 CONSORT and SPIRIT checklists.

Multimedia Appendix 2 Peer review report from Vice-Rector's Office for Research and Postgraduate Studies, Universidad Autónoma de Chile (translated from Spanish).

## Abbreviations

CONSORT: Consolidated Standards of Reporting Trials
RCT: randomized controlled trial
SPIRIT: Standard Protocol Items: Recommendations for Interventional Trials

## Data Availability

The datasets generated or analyzed during this study are available from the corresponding author on reasonable request.
