# Supplementary material for: Effects of a Walking-Based Physical Activity Intervention on Health Indicators in University Students: Protocol for a Randomized Controlled Trial
Source: JMIR Res Protoc. 2025 Dec 10;14:e83983. doi: 10.2196/83983 (PMC12739452; doi:10.2196/83983)
Supplement: Multimedia Appendix 1 [file resprot_v14i1e83983_app1.pdf]

Supplementary table 1. **CONSORT-Outcomes (for combined completion of CONSORT 2010 and CONSORT-Outcomes 2022 items)**[a]

Section	Item No.	CONSORT 2010 Item	CONSORT-Outcomes 2022 item	Location Reported[b]
**Title and abstract**				
	1a	Identification as a randomized trial in the title	-	1
	1b	Structured summary of trial design, methods, results, and conclusions (for specific guidance see CONSORT for abstracts)	-	1
**Introduction**				
Background and objectives	2a	Scientific background and explanation of rationale	-	1
	2b	Specific objectives or hypotheses	-	2
**Methods**				
Trial design	3a	Description of trial design (such as parallel, factorial) including allocation ratio	-	3
	3b	Important changes to methods after trial commencement (such as eligibility criteria), with reasons	-	3
Participants	4a	Eligibility criteria for participants	-	3
	4b	Settings and locations where the data were collected	-	3
Interventions	5	The interventions for each group with sufficient details to allow replication, including how and when they were actually administered (for specific guidance see TIDieR checklist and guide)	-	3
Outcomes	6a	Completely defined prespecified primary and secondary outcome measures, including how and when they were assessed	-	4
	6a.1		Provide a rationale for the selection of the domain for the trials primary outcome	4
	6a.2		Describe the specific measurement variable (eg, systolic blood pressure), analysis metric (eg, change from baseline, final value, time to event), method of aggregation (eg, mean,	5

© 2022 American Medical Association. All rights reserved.

Section	Item No.	CONSORT 2010 Item	CONSORT-Outcomes 2022 item	Location Reported[b]
			proportion), and the time point for each outcome	
	6a.3		If the analysis metric for the primary outcome represents within-participant change, define and justify the minimal important change in individuals	5
	6a.4		If the outcome data were continuous, but were analyzed as categorical (method of aggregation), specify the cutoff values used	5
	6a.5		If outcome assessments were performed at several time points after randomization, state the time points used for the analysis	5
	6a.6		If a composite outcome was used, define all individual components of the composite outcome	5
	6a.7		Identify any outcomes that were not prespecified in a trial registry or trial protocol	5
	6a.8		Provide a description of the study instruments used to assess the outcome (eg, questionnaires, laboratory tests) along with reliability, validity, and responsiveness in a population similar to the study sample	5
	6a.9		Describe who assessed the outcome (eg, nurse, parent) and any qualifications or trial-specific training necessary to administer the study instruments to assess the outcome	5
	6a.10		Describe any processes used to promote outcome data quality during data collection (eg, duplicate measurements) and after data collection (eg, range checks of outcome data values), or state where these details can be found	5
	6b	Any changes to trial outcomes after the trial commenced, with reasons	-	5
Sample size	7a	How sample size was determined	-	3

© 2022 American Medical Association. All rights reserved.

Section	Item No.	CONSORT 2010 Item	CONSORT-Outcomes 2022 item	Location Reported[b]
	7a.1		Define and justify the target difference between treatment groups (eg, the minimal important difference)	3
	7b	When applicable, explanation of any interim analyses and stopping guidelines	-	Not applicable
**Randomization**				
Sequence generation	8a	Method used to generate the random allocation sequence	-	3
	8b	Type of randomization; details of any restriction (such as blocking and block size)	-	3
Allocation concealment mechanism	9	Mechanism used to implement the random allocation sequence (such as sequentially numbered containers), describing any steps taken to conceal the sequence until interventions were assigned	-	3
Implementation	10	Who generated the random allocation sequence, who enrolled participants, and who assigned participants to interventions	-	3
Blinding	11a	If done, who was blinded after assignment to interventions (for example, participants, care providers, those assessing outcomes) and how	-	3
	11b	If relevant, description of the similarity of interventions	-	3
Statistical methods	12a	Statistical methods used to compare groups for primary and secondary outcomes	-	5
	12a.1		Describe any methods used to account for multiplicity in the analysis or interpretation of the primary and secondary outcomes (eg, coprimary outcomes, same outcome assessed at multiple time points, or subgroup analyses of an outcome)	5
	12a.2		State and justify any criteria for excluding any outcome data from the analysis and reporting, or report that no outcome data were excluded	6

© 2022 American Medical Association. All rights reserved.

Section	Item No.	CONSORT 2010 Item	CONSORT-Outcomes 2022 item	Location Reported[b]
	12a.3		Describe the methods used to assess patterns of missingness (eg, missing not at random), and describe the methods used to handle missing outcome items or entire assessments	6
	12a.4		Provide a definition of the outcome analysis population relating to nonadherence of the trial protocol (eg, as a randomized analysis)	6
	12b	Methods for additional analyses, such as subgroup analyses and adjusted analyses	-	6
**Results**				
Participant flow (a diagram is strongly recommended)	13a	For each group, the numbers of participants who were randomly assigned, received intended treatment, and were analyzed for the primary outcome	-	4
	13b	For each group, losses and exclusions after randomization, together with reasons	-	4
Recruitment	14a	Dates defining the periods of recruitment and follow-up	-	3
	14b	Why the trial ended or was stopped	-	3
Baseline data	15	A table showing baseline demographic and clinical characteristics for each group	-	Not applicable
Numbers analyzed	16	For each group, number of participants (denominator) included in each analysis and whether the analysis was by original assigned groups	-	Not applicable
Outcomes and estimation	17a	For each primary and secondary outcome, results for each group, and the estimated effect size and its precision (such as 95% CI)	-	Not applicable
	17a.1		Include the results for all prespecified outcome analyses or state where the results can be found if not in this report	Not applicable
	17b	For binary outcomes, presentation of both absolute and relative effect sizes is recommended	-	Not applicable
Ancillary analyses	18	Results of any other analyses performed, including subgroup analyses and adjusted	-	Not applicable

© 2022 American Medical Association. All rights reserved.

Section	Item No.	CONSORT 2010 Item	CONSORT-Outcomes 2022 item	Location Reported[b]
		analyses, distinguishing prespecified from exploratory		Not applicable
	18.1		If there were any analyses that were not prespecified, explain why they were performed	Not applicable
Harms	19	All important harms or unintended effects in each group (for specific guidance see CONSORT for harms)	-	Not applicabl e
**Discussion**				
Limitations	20	Trial limitations, addressing sources of potential bias, imprecision, and, if relevant, multiplicity of analyses	-	6
Generalizability	21	Generalizability (external validity, applicability) of the trial findings	-	6
Interpretation	22	Interpretation consistent with results, balancing benefits and harms, and considering other relevant evidence	-	6
**Other Information**				
Registration	23	Registration number and name of trial registry	-	5
Protocol	24	Where the full trial protocol can be accessed, if available	-	5
Funding	25	Sources of funding and other support (such as supply of drugs), role of funders	-	6

*A fillable version of the CONSORT Outcomes 2022 checklist can be found at http://www.consort statement.org

[a]It is strongly recommended that this checklist be read in conjunction with the CONSORT Outcomes and CONSORT Statement papers for important clarification on the items. The CONSORT Statement checklist is distributed under the terms of the Creative Commons Attribution License.

[b]Indicates page numbers and/or manuscript location: to be completed by authors.

Supplementary table 2.
SPIRIT 2013 Checklist: Recommended items to address in a clinical trial protocol and related documents*

Section/item	Item No	Description	Page
**Administrative information**			
Title	1	Descriptive title identifying the study design, population, interventions, and, if applicable, trial acronym	1
Trial registration	2a	Trial identifier and registry name. If not yet registered, name of intended registry	5
	2b	All items from the World Health Organization Trial Registration Data Set	-
Protocol version	3	Date and version identifier	1
Funding	4	Sources and types of financial, material, and other support	6
Roles and responsibilities	5a	Names, affiliations, and roles of protocol contributors	1, 6
	5b	Name and contact information for the trial sponsor	1
	5c	Role of study sponsor and funders, if any, in study design; collection, management, analysis, and interpretation of data; writing of the report; and the decision to submit the report for publication, including whether they will have ultimate authority over any of these activities	87
	5d	Composition, roles, and responsibilities of the coordinating centre, steering committee, endpoint adjudication committee, data management team, and other individuals or groups overseeing the trial, if applicable (see Item 21a for data monitoring committee)	7
**Introduction**			
Background and rationale	6a	Description of research question and justification for undertaking the trial, including summary of relevant studies (published and unpublished) examining benefits and harms for each intervention	1,2
	6b	Explanation for choice of comparators	2
Objectives	7	Specific objectives or hypotheses	2
Trial design	8	Description of trial design including type of trial (eg, parallel group, crossover, factorial, single group), allocation ratio, and framework (eg, superiority, equivalence, noninferiority, exploratory)	2,3
**Methods: Participants, interventions, and outcomes**			

Study setting	9	Description of study settings (eg, community clinic, academic hospital) and list of countries where data will be collected. Reference to where list of study sites can be obtained	3
Eligibility criteria	10	Inclusion and exclusion criteria for participants. If applicable, eligibility criteria for study centres and individuals who will perform the interventions (eg, surgeons, psychotherapists)	3
Interventions	11a	Interventions for each group with sufficient detail to allow replication, including how and when they will be administered	4
	11b	Criteria for discontinuing or modifying allocated interventions for a given trial participant (eg, drug dose change in response to harms, participant request, or improving/worsening disease)	4
	11c	Strategies to improve adherence to intervention protocols, and any procedures for monitoring adherence (eg, drug tablet return, laboratory tests)	4
	11d	Relevant concomitant care and interventions that are permitted or prohibited during the trial	4
Outcomes	12	Primary, secondary, and other outcomes, including the specific measurement variable (eg, systolic blood pressure), analysis metric (eg, change from baseline, final value, time to event), method of aggregation (eg, median, proportion), and time point for each outcome. Explanation of the clinical relevance of chosen efficacy and harm outcomes is strongly recommended	5
Participant timeline	13	Time schedule of enrolment, interventions (including any run-ins and washouts), assessments, and visits for participants. A schematic diagram is highly recommended (see Figure)	5
Sample size	14	Estimated number of participants needed to achieve study objectives and how it was determined, including clinical and statistical assumptions supporting any sample size calculations	3
Recruitment	15	Strategies for achieving adequate participant enrolment to reach target sample size	3
**Methods: Assignment of interventions (for controlled trials)**			
Allocation:			
Sequence generation	16a	Method of generating the allocation sequence (eg, computer-generated random numbers), and list of any factors for stratification. To reduce predictability of a random sequence, details of any planned restriction (eg, blocking) should be provided in a separate document that is unavailable to those who enrol participants or assign interventions	3

Allocation concealment mechanism	16b	Mechanism of implementing the allocation sequence (eg, central telephone; sequentially numbered, opaque, sealed envelopes), describing any steps to conceal the sequence until interventions are assigned	3
Implementation	16c	Who will generate the allocation sequence, who will enrol participants, and who will assign participants to interventions	4
Blinding (masking)	17a	Who will be blinded after assignment to interventions (eg, trial participants, care providers, outcome assessors, data analysts), and how	4
	17b	If blinded, circumstances under which unblinding is permissible, and procedure for revealing a participant's allocated intervention during the trial	4
**Methods: Data collection, management, and analysis**			
Data collection methods	18a	Plans for assessment and collection of outcome, baseline, and other trial data, including any related processes to promote data quality (eg, duplicate measurements, training of assessors) and a description of study instruments (eg, questionnaires, laboratory tests) along with their reliability and validity, if known. Reference to where data collection forms can be found, if not in the protocol	5
	18b	Plans to promote participant retention and complete follow-up, including list of any outcome data to be collected for participants who discontinue or deviate from intervention protocols	5
Data management	19	Plans for data entry, coding, security, and storage, including any related processes to promote data quality (eg, double data entry; range checks for data values). Reference to where details of data management procedures can be found, if not in the protocol	5
Statistical methods	20a	Statistical methods for analysing primary and secondary outcomes. Reference to where other details of the statistical analysis plan can be found, if not in the protocol	5
	20b	Methods for any additional analyses (eg, subgroup and adjusted analyses)	6
	20c	Definition of analysis population relating to protocol non-adherence (eg, as randomised analysis), and any statistical methods to handle missing data (eg, multiple imputation)	6
**Methods: Monitoring**			
Data monitoring	21a	Composition of data monitoring committee (DMC); summary of its role and reporting structure; statement of whether it is independent from the sponsor and competing interests; and reference to where further details about its charter can be found, if not in the protocol. Alternatively, an explanation of why a DMC is not needed	6

	21b	Description of any interim analyses and stopping guidelines, including who will have access to these interim results and make the final decision to terminate the trial	6
Harms	22	Plans for collecting, assessing, reporting, and managing solicited and spontaneously reported adverse events and other unintended effects of trial interventions or trial conduct	6
Auditing	23	Frequency and procedures for auditing trial conduct, if any, and whether the process will be independent from investigators and the sponsor	6
**Ethics and dissemination**			
Research ethics approval	24	Plans for seeking research ethics committee/institutional review board (REC/IRB) approval	3
Protocol amendments	25	Plans for communicating important protocol modifications (eg, changes to eligibility criteria, outcomes, analyses) to relevant parties (eg, investigators, REC/IRBs, trial participants, trial registries, journals, regulators)	3
Consent or assent	26a	Who will obtain informed consent or assent from potential trial participants or authorised surrogates, and how (see Item 32)	3
	26b	Additional consent provisions for collection and use of participant data and biological specimens in ancillary studies, if applicable	3
Confidentiality	27	How personal information about potential and enrolled participants will be collected, shared, and maintained in order to protect confidentiality before, during, and after the trial	7
Declaration of interests	28	Financial and other competing interests for principal investigators for the overall trial and each study site	7
Access to data	29	Statement of who will have access to the final trial dataset, and disclosure of contractual agreements that limit such access for investigators	7
Ancillary and post-trial care	30	Provisions, if any, for ancillary and post-trial care, and for compensation to those who suffer harm from trial participation	7
Dissemination policy	31a	Plans for investigators and sponsor to communicate trial results to participants, healthcare professionals, the public, and other relevant groups (eg, via publication, reporting in results databases, or other data sharing arrangements), including any publication restrictions	7
	31b	Authorship eligibility guidelines and any intended use of professional writers	7
	31c	Plans, if any, for granting public access to the full protocol, participant-level dataset, and statistical code	7

Appendices

Informed consent materials	32	Model consent form and other related documentation given to participants and authorised surrogates	7
Biological specimens	33	Plans for collection, laboratory evaluation, and storage of biological specimens for genetic or molecular analysis in the current trial and for future use in ancillary studies, if applicable	-

*It is strongly recommended that this checklist be read in conjunction with the SPIRIT 2013 Explanation & Elaboration for important clarification on the items. Amendments to the protocol should be tracked and dated. The SPIRIT checklist is copyrighted by the SPIRIT Group under the Creative Commons "Attribution-NonCommercial-NoDerivs 3.0 Unported" license.
