# Supplementary material for: Effects of a Walking-Based Physical Activity Intervention on Health Indicators in University Students: Protocol for a Randomized Controlled Trial
Source: JMIR Res Protoc. 2025 Dec 10;14:e83983. doi: 10.2196/83983 (PMC12739452; doi:10.2196/83983)
Supplement: Multimedia Appendix 2 [file resprot_v14i1e83983_app2.pdf]

Paola Fuentes Merino.
PRESENT

Santiago, Chile, September 3, 2025

On behalf of the Research Directorate, you are given the grades obtained for your research project submitted to the "Research Support 2025" contest, entitled: **"Effect of a walking-based physical activity intervention on health indicators in university students from Santiago, Talca and Temuco. A randomized controlled trial."**
The maximum score achieved in this call was **4,920** and the last selected project obtained a score of **4,258**. Their application obtained a score of **4,750** and the **8th place** among the 65 applications admissible to the contest.

Reviewer 1

Notes
Theoretical foundation: 5
Methodology: 5
Feasibility: 5
Impact and newness: 5

Feedback

Quality of the proposal.
The research proposal presented is relevant to the extent that it is a relevant topic for the country. The project is well written and has a solid theoretical basis that enriches the hypotheses described.
As for the methodology, presenting a randomized trial enriches the proposal, increasing the quality of the research.
The researcher is suggested to present more information on the activities that the control group will do and the characteristics of the pedometer to be used. In addition, justify the time of the intervention and explain how they will control that the participants do not perform physical activities outside the intervention, which may affect the results.

Viability.
The project is viable, because the relevant aspects of the intervention are described. As it is a walking intervention, it does not have risk or difficulty in attracting the participants.

Impact and scientific novelty.
This type of study has a high impact, since it will benefit the university population. Various international bodies have issued a call to promote this type of intervention in the general population.

Reviewer 2

Notes
Theoretical basis: 4.5
Methodology: 4.5
Viability: 4.5
Impact and newness: 4.5

Hoping that these comments will be useful, without any other particular, he bids farewell cordially,

DIRECCIÓN DE INVESTIGACIÓN
UNIVERSIDAD AUTONOMA DE CHILE

Dra. Ana Gutiérrez Moraga
Directora de Investigación
Vicerrectoría de Investigación y Doctorados
